# Peptaibols from *Trichoderma asperellum* TR356 strain isolated from Brazilian soil

**DOI:** 10.1186/2193-1801-3-600

**Published:** 2014-10-13

**Authors:** João PC Brito, Marcelo HS Ramada, Mariana TQ de Magalhães, Luciano P Silva, Cirano J Ulhoa

**Affiliations:** Departamento de Bioquímica e Biologia Molecular, Universidade Federal de Goiás (ICB II), 74001-970 Goiânia, GO Brasil; Empresa Brasileira de Pesquisa Agropecuária, Centro Nacional de Pesquisa de Recursos Genéticos e Biotecnologia, PBI. Parque Estação Biológica, Asa Norte, 70910-90 Brasília, DF Brasil; Department of Molecular, Microbial and Structural Biology, University of Connecticut Health Center – Uconn Health Center, 263 Farmington Avenue, Farmington, CT 06030 USA

**Keywords:** *Trichoderma asperellum*, Peptaibols, Mass spectrometry, Asperelines, Trichotoxins

## Abstract

The *Trichoderma* genus consists of a group of free-living filamentous fungi, including species able to act as biological control agents (BCAs) against pathogenic fungi. It is believed that this ability is due to synergy between several mechanisms, including the production of a wide variety of secondary metabolites by these organisms. Among these, we highlight the production of peptaibols, an antibiotic peptide group characterized by the presence of non-proteinogenic amino acids such as α-aminoisobutyrate (Aib), as well as by N-terminal modifications and amino alcohols in the C-terminal region. This study aimed to outline a profile of peptaibol production and to identify secreted peptaibols from the *Trichoderma asperellum* TR356 strain, described as an efficient BCA against *S. sclerotiorum*. The fungus was grown on TLE 0.3% glucose medium for 5 days, with agitation at 120 rpm in the dark. Liquid medium filtrate was used as the metabolite source. These extracts were subjected to high performance liquid chromatography (HPLC) and subsequent analysis by matrix-assisted laser desorption ionization mass spectrometry (MALDI-TOF). The results indicate the production of two classes of peptaibols for this *T. asperellum* strain. Primary structures of two asperelines (A and E) and five trichotoxins (T5D2, T5E, T5F, T5G and 1717A) have been elucidated. Most of these peptaibols had been previously described in *T. viride* and *T. asperellum* marine strains. This is the first report of some of these compounds being produced by a *T. asperellum* strain from soil. Future analyses will be necessary to elucidate the three-dimensional structures and their activities against pathogens.

## Introduction

The *Trichoderma* genus belongs to the Ascomycota phylum, Hypocrales order, Hypocreaceae family and corresponds to filamentous fungi that are free-living and widely distributed in nature. One of the main characteristics of this genus is its ability to act as a biological control agent (BCA) against phytopathogenic microorganisms such as *Sclerotinia sclerotiorum*, *Rhizoctonia solani* and *Fusarium* spp. (Harman, 2006; Schuster & Schmoll, 2010).

The biological control mechanisms can be divided into direct and indirect effects. Direct effects include nutrient and space competition, volatile and non-volatile antibiotic production, hydrolytic enzyme production and parasitism. Indirect effects include all aspects that promote morphological and biochemical changes in the host plant, such as nutrient availability, stress responses and induction of resistance to diseases caused by plant pathogens (Harman *et al.*, 2004; Vinale *et al.,*^2008^; Vinale *et al.,*^2012^). It is believed that *Trichoderma’s* extensive mycoparasitism ability is also related to the wide variety of secondary metabolites produced by these organisms (O’Brian & Wright, 2011; Mukherjee *et al.*, 2012).

Among secondary metabolites produced by *Trichoderma* spp., there are reports and descriptions of such compounds as pyrones, terpenoids, steroids, gliotoxins, gliovirins (Reino *et al.*, 2008), and some antibiotic peptides, known as peptaibols (Daniel, 2007). The discovery of peptide antibiotics produced by fungi has attracted attention because they represent a possible weapon against pathogens. Peptaibols are included in a class of compounds called peptaibiotics. They are defined as peptides derived from fungal secondary metabolism, consisting of 4 to 21 amino acid residues approximately. One of their main features is the presence of non-proteinogenic amino acids such as α-aminoisobutyrate acid (Aib), isovaline (Iva), ethylnorvaline (EtNor) and hydroxyproline (Hyp). (Stoppacher *et al.*, 2013). At the N-terminus there are modifications such as acyl or acetyl groups, and at the C-terminus there is the presence of an amino alcohol such phenylalaninol (Phe-OH), prolinol (Pro-OH) or valinol (Val-OH) (Daniel, 2007). The name "peptaibol" is derived from *pep*tide, *Aib* and amino alcoh*ol*, referring to these main features.

Several peptaibols from Trichoderma have already been described. Recently, "The Comprehensive Peptaibiotics Database" was created, including information such as amino acid sequences, molecular formulae, monoisotopic masses, and groupings for 1043 peptaibiotics (Stoppacher *et al.*, 2013; http://peptaibiotics-database.boku.ac.at). Because some of these compounds present antimicrobial activity, there is a growing interest in their identification and structure elucidation. Peptaibols from *Trichoderma* have been reported as showing activity against mycoplasma (Beven *et al.*, 1998), *Staphylococcus aureus* and *Sclerotium rolfsii* (Rebuffat et al. 1995). Some peptaibols, called trichorzianins, acted in synergy with *T. harzianum* chitinases and β-1,3-glucanases against *Botrytis cinerea*, and it was reported that these same peptaibols inhibited the activity of *B. cinerea* β-1,3-glucan synthases, preventing cell wall reconstruction (Schirmböck *et al.*, 1994; Lorito *et al.,*^1996^).

There are also reports of *T. harzianum* peptaibols causing biological changes in human lung epithelial carcinoma cells (Peltola et al. 2004), and of *T. koningii* peptaibols inhibiting hepatocellular carcinoma cell growth by causing an increase in calcium influx and consequent activation of the apoptosis pathway (Wang *et al.,*^2010^). Some studies suggest that their biological activity is related to their amphipathic nature, allowing these compounds to form ion channels in lipid membranes (Mikkola *et al.,*^2012^; Rahaman & Lazaridis, 2014). Some studies have focused on peptaibols produced by *Trichoderma* in genus classification and taxonomy (Degenkolb *et al.*, 2008).

Peptaibols fit into a group of metabolites called non-ribosomal peptides. This is because these compounds do not result from gene transcription and subsequent translation, but are formed from multi-enzyme complex called Non-Ribosomal Peptide Synthetases (NRPSs). In summary, these multienzyme complexes are formed by a set of modules, where each module has catalytic domains responsible for the synthesis steps (Schwarzer *et al.*, 2003). In general, there are three main steps, starting in domain A (adenylation), where the biosynthetic process begins with amino acid entry, which is activated by adenylation. The activated amino acid is attached to a PCP protein cofactor (HS-4′PP) that acts as a carrier between the catalytic centers, and finally, in domain C (condensation), peptide bond formation occurs (Mootz *et al.*, 2002; Schwarzer *et al.*, 2003).

In this context, this study aimed to draw a production profile and identify peptaibols secreted by the *Trichoderma asperellum* TR356 strain, based on a recent study conducted by Geraldine and colleagues (2013), showing that this strain had high efficiency in the control of white mold, a disease caused by the pathogen *Sclerotinia sclerotiorum*. In that study the authors attributed the biological control efficiency to the high production of hydrolytic enzymes such as NAGase and β-1,3-glucanase, involved in host cell wall degradation. However, the involvement of other secondary metabolites such as peptaibols, was not analyzed. In the literature there are several reports of peptaibols identified from strains of *T. asperellum*, but the production mechanisms of these compounds between strains are not identical (Chutrakul *et al.*, 2008; Ren *et al.*, 2009; Ren *et al.*, 2013). Based on this, we determined the need to investigate which peptides are produced by *T. asperellum* TR356 strain, a strain that has been isolated in Brazil.

## Method

### Strain maintenance and spore solution

For *Trichoderma* strain maintenance, 3 mm diameter discs originating from culture plates were periodically transferred to new Petri dishes containing MYG medium [5 g.L^-1^ of malt extract (Biobrás), 2.5 g.L^-1^of yeast extract (Acumedia), 10 g.L^-1^ of glucose (Sigma-Aldrich), and 20 g.L^-1^ of agar (Kasvi)]. After sporulation, the spores were stored in 0.9% (w/v) saline solution. All strains were donated by EMPRAPA-CNPAF and ICB/UFG Enzymology Laboratory collection.

### Induction

For peptaibol production, approximately 10^7^*Trichoderma* spores were added to a 500 mL Erlenmeyer flask containing 200 mL of TLE medium [CaCl_2_.2H_2_O 0.3 g.L^-1^ (Sigma-Aldrich), KH_2_PO_4_ 2.0 g.L^-1^ (Vetec), (NH_4_)_2_SO_4_ 1.4 g.L^-1^ (Vetec), MgSO_4_.7H_2_O 0.3 g.L^-1^ (Vetec), urea 0.3 g.L^-1^ (Vetec), peptone 1.0 g.L^-1^ (MicroMed), trace elements solution (Fe^2+^, Zn^2+^, Mn^2+^, Cu^2+^) 0.1% (v/v) and glucose 0.3% (w/v, Sigma-Aldrich). The flasks were incubated on a rotary shaker at 28°C, with 120 rpm in the dark for 5 days.

### Extraction

After induction, the medium was separated from mycelia by vacuum filtration, and the peptides were extracted by adding ethyl acetate (1:2 v/v, Dinâmica). After phase separation in a separating funnel, the apolar phase was collected, centrifuged and rotoevaporated. The residue formed was resuspended by washing with MilliQ water and then with acetonitrile (J.T. Baker), and collected separately. The acetonitrile fraction was lyophilized and used as a peptaibol source.

### Liquid chromatography

The lyophilized material was resuspended in acetonitrile 5% (w/v), with final concentration of 200 mg.ml^-1^, and subjected to high performance liquid chromatography in reverse phase (HPLC) on an analytical Vydac Protein & Peptide C18 platform. The eluents were A) MilliQ water with 0.1% TFA (v/v) and B) acetonitrile with 0.1% TFA (v/v). Flow rate of 1 mL/min. The concentration gradient started with 5% B (v/v) for 5 min., increased from 5% (v/v) to 95% (v/v) during 40 min. and finished in 95% (v/v). Detection was performed at 216 and 280 nm. The observed peaks were manually collected in 1.5 mL microtubes. Chromatograms were obtained by Shimadzu Class VP software.

### Mass spectrometry

The mass spectrometric analysis was performed by MALDI-TOF-TOF (UltraFlex III, Bruker Daltonics, Bremen, Germany) controlled by FlexControl 3.0 software (Bruker Daltonics). The fractions collected from HPLC were mixed with an α-acid matrix (cyano-4-hydroxycinnamic acid matrix) solution, 10 mg/mL (1:3) on an AnchorChip MTP 400/384 Mass (Bruker Daltonics) plate and dried at room temperature. The acquisition of MS spectra was performed at *m/z* 120 to 2500, reflected and positive mode, with external calibration (Protein Calibration Standart, Bruker). Peptaibol MS/MS spectra were obtained by means of LIFT fragmentation after analyzing the MS spectra and selection of precursor ions for fragmentation. For data analysis the FlexControl 3.0 software (Bruker Daltonics) was used. These steps were carried out at the Laboratory of Mass Spectrometry, CENARGEN/EMBRAPA, Brasilia, DF, Brazil.

## Results and discussion

**Figure 1:**
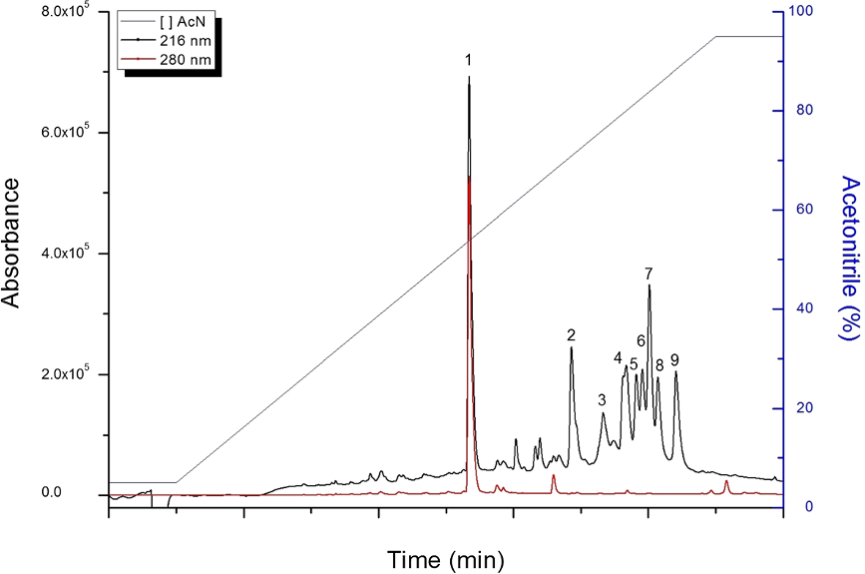
***T. asperellum*** **TR356 extract chromatography (HPLC).** Detection at 216 nm (black) and 280 nm (red). The gradient of acetonitrile + 0.1% TFA is shown in blue.

From the *T. asperellum* TR356 extract chromatogram, 9 fractions were detected after 26.7 min (Figure 1). This shows a hydrophobic profile for these compounds, as shared by most peptaibiotics. Fractions were manually collected and analyzed by MALDI-TOF-TOF. No peptaibols were detected in fraction 1.

**Figure 2:**
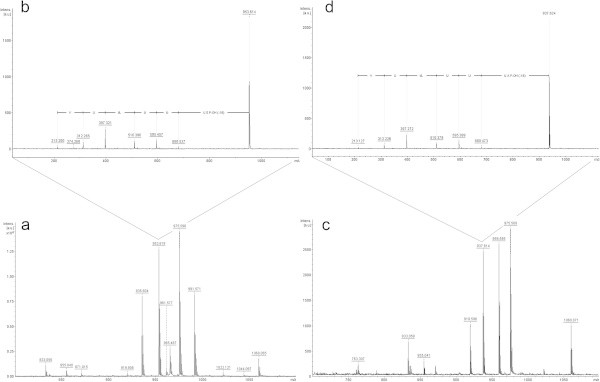
**Positive-ion MS from** ***T. asperellum*** **TR356 fractions 2 (a) and 3 (c); Also shown is the fragmentation of the ion with** ***m/z*** **953.6 from fraction 2 (b) and** ***m/z*** **937.6 from fraction 3 (d).**

Fraction 2 analysis revealed the presence of many different ions, of which the most prominent were those with *m/z* 935.6 [M + H^+^], 953.6 [M + H^+^], 975.5 [M + Na^+^] and 991.5 [M + H^+^] (Figure 2a). Fragmentation of the latter two and interpretation of the first were not possible. Analyzing the mass spectrum of the ion with *m/z* 953.6 [M + H^+^] (Figure 2b), we can see a partial sequence of five amino acids (Val - Aib - Lxx - Aib - Aib), and a difference of 213 Da at the N-terminus and 273 Da at the C-terminus. Referring to the "The Comprehensive Peptaibiotics Database", an ion with *m/z* 952.6 was identified as an Asperelin E (Ren *et al.,*^2009^). From this reference then it is assumed that the 213 Da difference observed in this spectrum is related to two Aib residues, one of them with an acetyl group. Likewise, the 273 Da difference corresponds to Aib, serine and prolinol residues. Finally, a difference of 18 Da is related to loss of a water molecule.

In fraction 3, the most prominent ions were those with *m/z* 919.6 [M + H^+^], 937.6 [M + H^+^], 959.6 [M + Na^+^] and 975.5 [M + H^+^] (Figure 2c). As with the previous fraction, the fragmentation of the last two ions, and interpretation of the first one was not possible. In the analysis of the ion with *m/z* 937.7 [M + H^+^], a partial sequence was observed (Val - Aib - Lxx - Aib - Aib) (Figure 2d), which was the same as the ion with *m/z* 953.6 [M + H^+^] in the previous fraction. We also observed the difference of 213 Da at the N-terminus and 257 Da at the C-terminus. Referring to the database, it was observed that Asperelin A, also described by Ren *et al.* (2009), has a similar mass (936.6 Da), suggesting the presence of this compound in this fraction. Again using this reference, it is assumed that the difference of 213 Da corresponds to two Aib residues, one of them with an acetyl group, and the difference of 257 Da in the C-terminal portion seems to be referring to an alanine, Aib and prolinol (as well as 18 Da for the water molecule).

The Asperelin group was previously described only in *Trichoderma asperellum* marine strains (Ren *et al.*, 2009; Ren *et al.*, 2013). We believe that this is the first description of these compounds being produced by a soil strain of *Trichoderma asperellum*.

**Figure 3:**
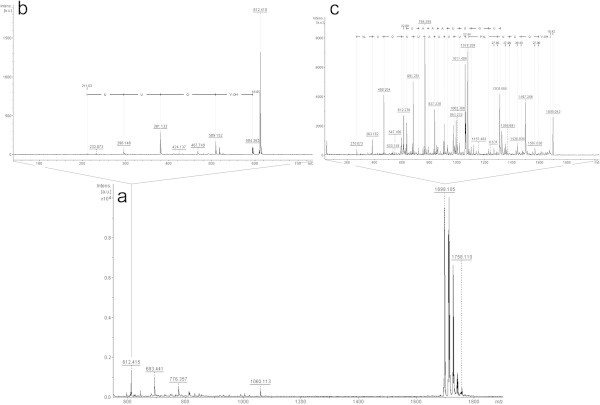
**Positive-ion MS from** ***T. asperellum*** **TR356 fraction 4 (a); Fragmentation of ions** ***m/z*** **1698.1 (c) and** ***m/z*** **612 (b).**

In the fraction 4 analysis (Figure 3a), interpretation of just two ions were possible. The ion with *m/z* 1698.1 [M + Na^+^] (Figure 3c) showed most of b-series and part of the y-series. In the N-terminal portion of the b-series there is a difference of 270 Da, and between isovaline and proline, there is a sodium ion, also observed in the y-series. In the C-terminal portion of the y-series it is possible to find an ion of *m/z* 612. In Jaworski & Brückner (1999) *T. viride* trichotoxins study, it was noted that the bond between Aib and proline on these peptaibols is extremely labile, thus generating two ions resulting from this break.

This feature would explain the binding of sodium ions in this exact position in both series, and the presence of the *m/z* 612 ion, which can also be observed in the spectrum of fraction 4 (Figure 3a). After fragmentation, this ion seems to confirm what had been observed in the b-series (Figure 3b). The difference of 211 Da suggested the presence of a proline and leucine/isoleucine. After comparing the sequence to those in the databases, it was possible to identify a peptaibol named trichotoxin T5D2 with molecular weight of 1675 Da (Suwan *et al.*, 2000). From this reference then we can assume that the difference of 270 Da corresponds to an Aib + acetyl group, a glycine, and a second Aib. The trichotoxin T5D2 identified in this spectrum has not yet been described in *T. asperellum*.

**Figure 4:**
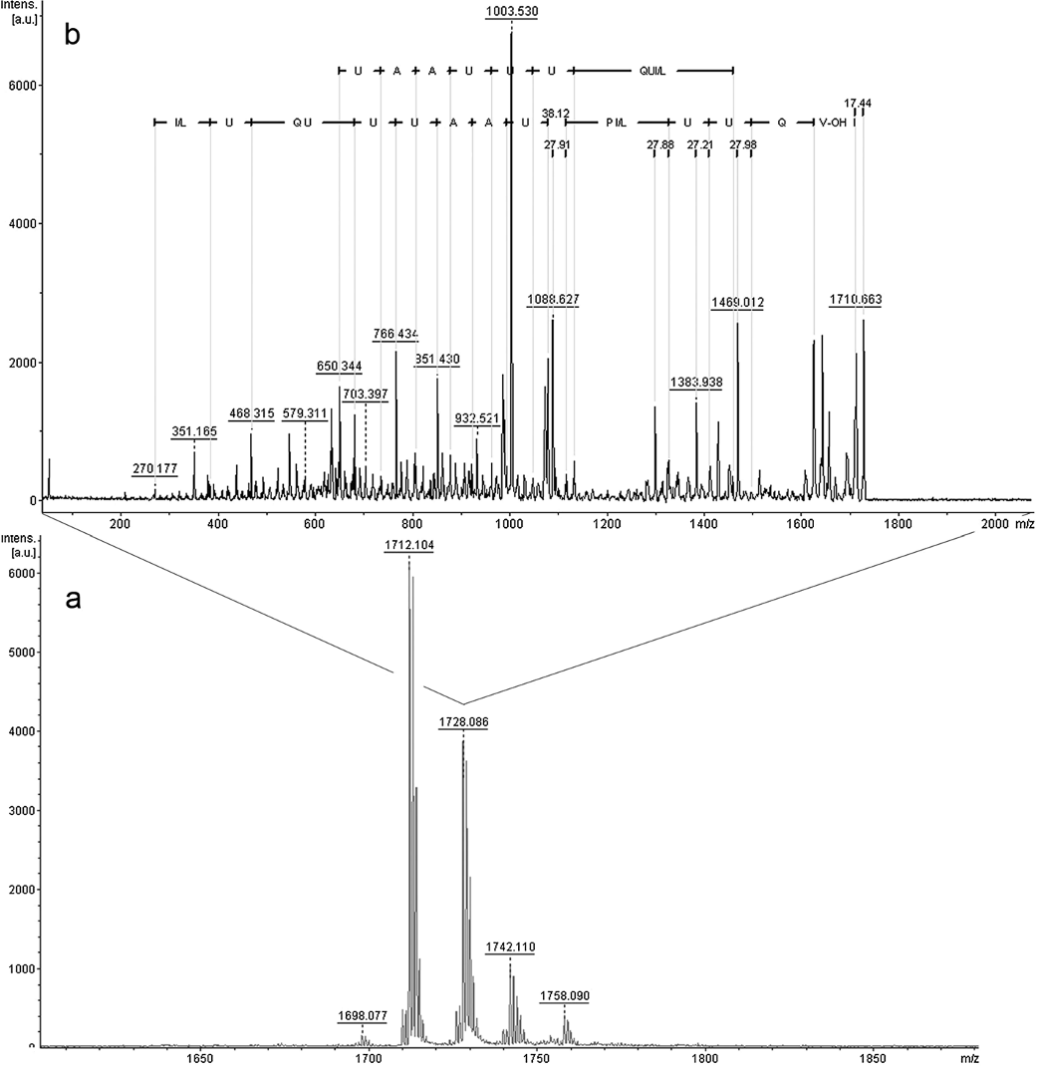
**Positive-ion MS from** ***T. asperellum*** **TR356 fraction 5 (a); Fragmentation of ion** ***m/z*** **1728.0 (b).**

In fraction 5 (Figure 4a) only an ion of *m/z* 1728.0 [M + K^+^] was interpretable (Figure 4b). Interpretation of the ion with *m/z* 1712.1 was not possible because there seemed to be more than one peptide being fragmented together. Again, it is possible to see part of y-series, most of the b-series, and a difference of 270 Da at the beginning of the b-series. We also noted the presence of a potassium ion between Aib and proline due to the fragility of that bond, as discussed previously. In this fraction, the ion with m/z 612 was also seen (data not shown), and when fragmented this has the same sequence as that described above. Sequence analysis suggests that this peptaibol is a trichotoxin A-50 E (T5E), first identified by Bruckner & Przybylski (1984) and isolated from *T. viride*. From this information, it is assumed that the difference of 270 Da corresponds to Aib + acetyl group, glycine, and a second Aib. This peptaibol has not previously been described in isolates of *T. asperellum*.

**Figure 5:**
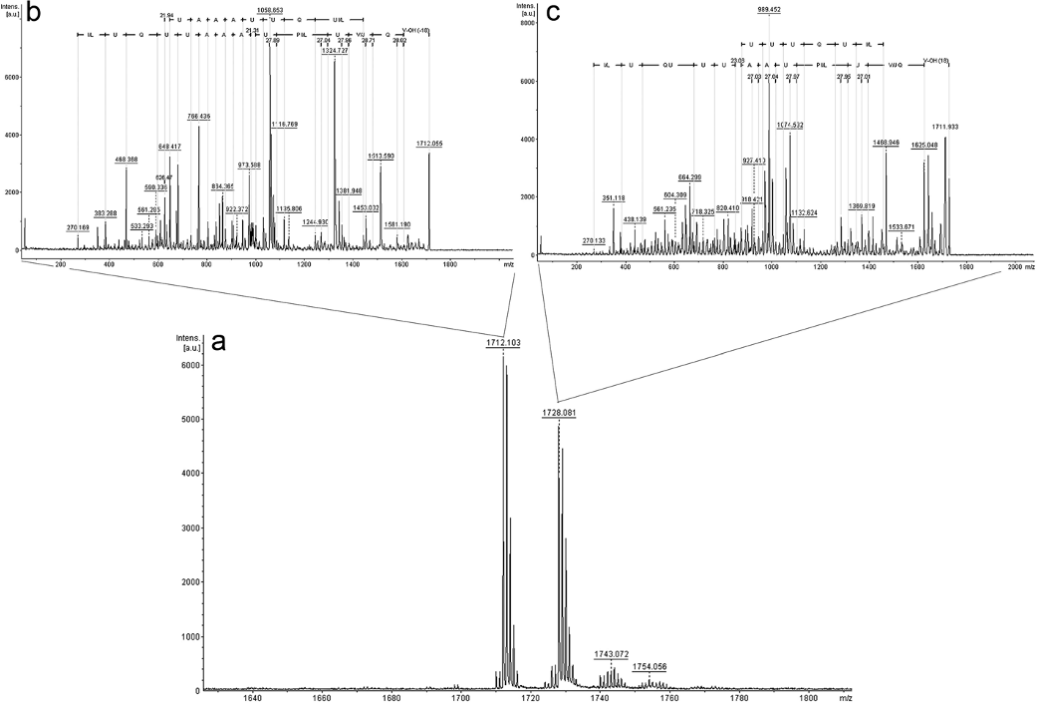
**Positive-ion MS from** ***T. asperellum*** **TR356 fraction 6 (a); Fragmentation of ions** ***m/z*** **1712.1 (b) and** ***m/z*** **1728.0 (c).**

In the analysis of fraction 6, the two most prominent features are ions of *m/z* 1712.1 [M + Na^+^] and 1728.0 [M + Na^+^] (Figure 5a). In the *m/z* 1712.1 [M + Na^+^] ion fragmentation we observed part of the y-series and most of the b-series. Again, as reported previously, there is a difference of 270 Da in the beginning of the b- series, and the presence of sodium ions, which in this analysis are in different positions in each series (Figure 5b). The Y-series appears to finish at *m/z* 626.4, probably due to the labile bond between proline and Aib as discussed above. Thus, when we look at the fraction 6 mass spectrum in this region, it is possible to detect and fragment this ion, confirming part of the series that was not observed in the *m/z* 1712.1 [M + Na^+^] fragmentation. Based on these data, the analysis of this sequence was similar to the trichotoxin A-50 F (T5F) also described by Bruckner & Przybylski (1984) in *T. viride*.

Fragmentation of the *m/z* 1728.0 ion elucidated only a small part of y-series, and in the b-series there is the presence of a sodium ion between Aib and alanine residues (Figure 5c). This sequence, when compared to the databases is similar to trichotoxin A-50 G (T5G), also identified by Bruckner & Przybylski (1984). Neither of the peptaibols found in this fraction (T5F and T5G) have been previously described as being produced by *T. asperellum* strains.

**Figure 6:**
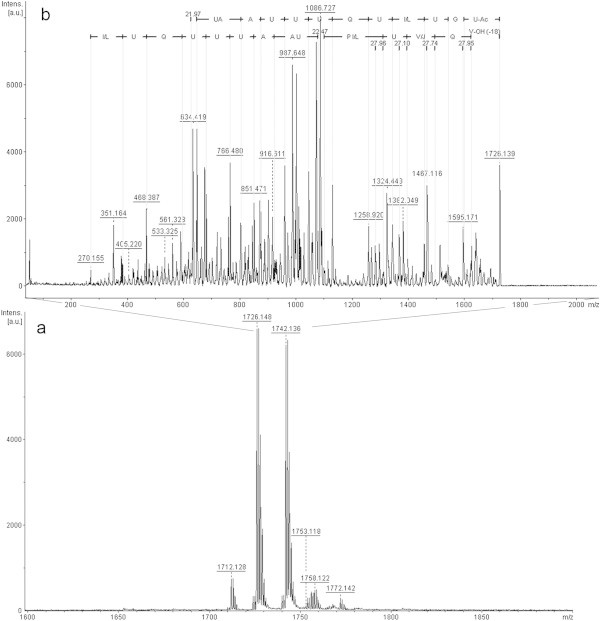
**Positive-ion MS from** ***T. asperellum*** **TR356 fraction 7 (a); Fragmentation of ion** ***m/z*** **1726.1 (b).**

**Figure 7:**
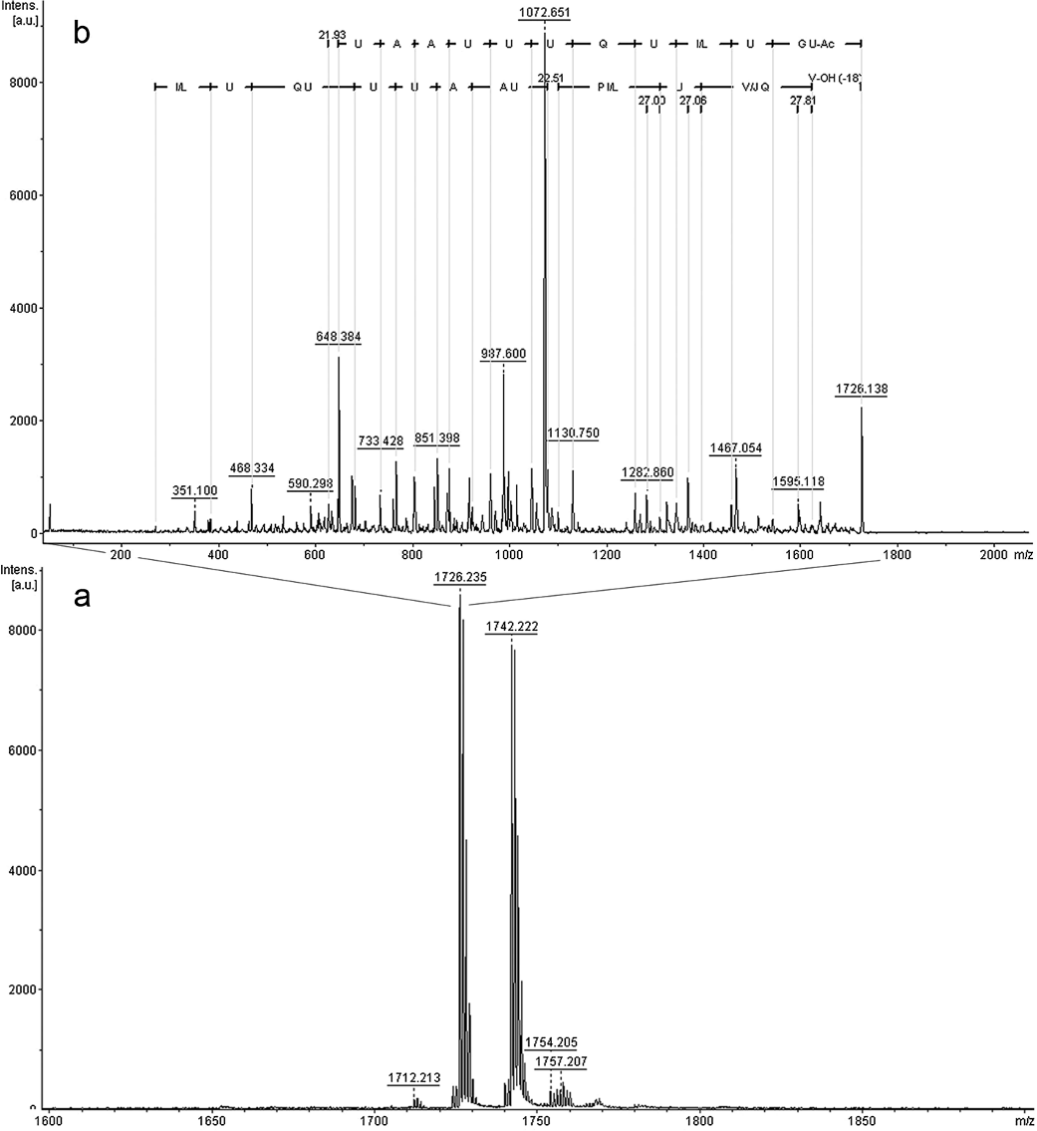
**Positive-ion MS from** ***T. asperellum*** **TR356 fraction 8 (a); Fragmentation of ion** ***m/z*** **1726.2 (b).**

In fraction 7, two prominent ions were observed (Figure 6a), but only the *m/z* 1726.1 [M + Na^+^] profile was elucidated. On this fragmentation mass spectrum (Figure 6b), we can see part of both series (-y and -b). Again we see the presence of a sodium ion between Aib and proline residues, and in this mass spectrum it was possible to identify the amino acid residues present in the y- series N-terminus, confirming what we find in literature. The analysis of fraction 8 (Figure 7a) was identical to fraction 7, and again only the ion of *m/z* 1726.2 [M + Na^+^] was interpreted (Figure 7b). By comparing these sequences with the databases, in both fractions there are peptaibols similar to the trichotoxin A-50 G (T5G), also found in the previous fraction.

**Figure 8:**
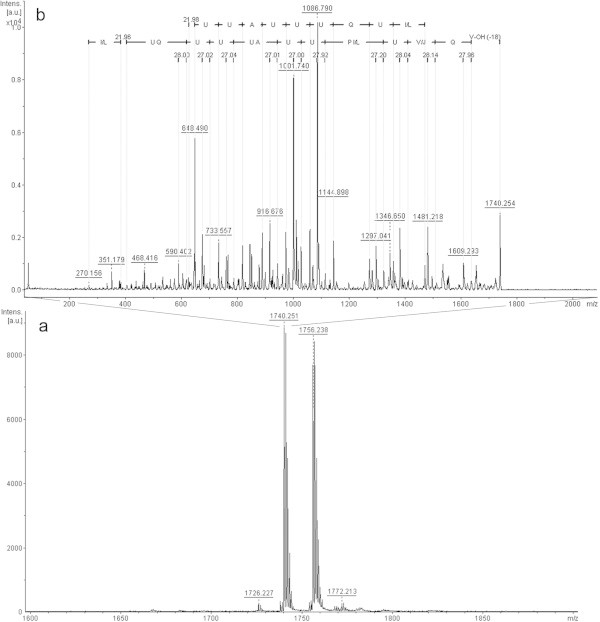
**Positive-ion MS from** ***T. asperellum*** **TR356 fraction 9 (a); Fragmentation of ion** ***m/z*** **1740.2 (b).**

In the last fraction collected from the *T. asperellum* TR356 extract (Figure 8a), two ions were observed, and only the ion with m/z 1740.2 [M + Na^+^] was interpreted (Figure 8b). In this MS we observed the presence of sodium ions between Aib and proline in the y-series, and between leucine/isoleucine and Aib in the b-series. This sequence is similar to trichotoxin 1717A (Chutrakul *et al*. 2008), isolated from *T. asperellum* strains.

The trichotoxins are a group of approximately 27 peptaibols. They were initially identified by Brückner & Przybylski (1984) from isolates of *T. viride*, and subsequently further characterized by Jaworski & Brückner (1999). Later, new trichotoxins were identified from *T. harzianum* (Suwan *et al.*, 2000) and *T. asperellum* strains (Chutrakul *et al.*, 2008), who demonstrated for the first time their antimicrobial activity against *B. stearothermophilus*.

Tables 1 and 2 summarize the information obtained from each *T. asperellum* TR356 fraction analysis.

**Table 1:** **Peptaibol sequences from** ***T. asperellum*** **TR356 strain extract; Mod.: N-terminal modification; Lxx: leucine or isoleucine; Vxx: valine or isovaline**

Peptaibol	Mod.	1	2	3	4	5	6	7	8	9	10	11	12	13	14	15	16	17	18
Asperelin E	Ac	Aib	Aib	Val	Aib	Lxx	Aib	Aib	Ser	Aib	Prool								
Asperelin A	Ac	Aib	Aib	Val	Aib	Lxx	Aib	Aib	Ala	Aib	Prool								
Trichotoxin T5D2	Ac	Aib	Gly	Aib	Lxx	Aib	Gln	Aib	Aib	Ala	Ala	Ala	Aib	Pro	Lxx	Aib	Aib	Gln	Valol
Trichotoxin A-50 E (T5E)	Ac	Aib	Gly	Aib	Lxx	Aib	Gln	Aib	Aib	Aib	Ala	Ala	Aib	Pro	Lxx	Aib	Aib	Gln	Valol
Trichotoxin A-50 F (T5F)	Ac	Aib	Gly	Aib	Lxx	Aib	Gln	Aib	Aib	Ala	Ala	Ala	Aib	Pro	Lxx	Aib	Vxx	Gln	Valol
Trichotoxin A-50 G (T5G)	Ac	Aib	Gly	Aib	Lxx	Aib	Gln	Aib	Aib	Aib	Ala	Ala	Aib	Pro	Lxx	Aib	Vxx	Gln	Valol
Trichotoxin 1717A	Ac	Aib	Gly	Aib	Lxx	Aib	Gln	Aib	Aib	Aib	Ala	Aib	Aib	Pro	Lxx	Aib	Vxx	Gln	Valol

**Table 2:** **Further information regarding** ***T. asperellum*** **TR356 strain peptaibols; RT: chromatography retention time**

Peptaibol	***m/z***	Fraction	RT (min)
Asperelin E	953,6 [M + H^+^]	2	34,3
Asperelin A	937,6 [M + H^+^]	3	36,6
Trichotoxin T5D2	1698,0 [M + Na^+^]	4	38,4
Trichotoxin A-50 E (T5E)	1728,0 [M + K^+^]	5	39,1
Trichotoxin A-50 F (T5F)	1712,0 [M + Na^+^]	6	39,5
Trichotoxin A-50 G (T5G)	1726,1 [M + Na^+^]	6 | 7 | 8	39,5 | 40,1 | 40,7
Trichotoxin 1717A	1740,2 [M + Na^+^]	9	42

## Conclusion

The genus *Trichoderma* is known to act as an ACB through several mechanisms including the production of peptaibols. Among various species, the *T. asperellum* TR356 strain was recently described as an efficient ACB against *S. sclerotiorum* in the field, but the production of secondary metabolites had not been previously reported. Using data obtained in this study, it was concluded that *T. asperellum* TR356 strain is able to produce different peptaibols, such as asperelins A and E, and trichotoxins T5D2, 1717A and A-50 E, F and G. Many of these peptaibols have already been described as being produced by *T. asperellum* strains and other *Trichoderma* species; however, this is the first report of these compounds being produced by a *T. asperellum* strain isolated in Brazil. It was not possible to measure the antimicrobial activity of these peptaibols here, since production levels were insufficient for such tests. Further analysis will be needed to elucidate the involvement of these compounds in the *Trichoderma* biological control process against plant pathogens, and to elucidate the three-dimensional structures of these molecules.

## References

[CR1] Béven L, Duval D, Rebuffat S, Riddell FG, Bodo B, Wróblewski H (1998). Membrane permeabilisation and antimycoplasmic activity of the 18-residue peptaibols, trichorzins PA. Biochem Biophysi Acta.

[CR2] Brückner H, Przybylski M (1984). Isolation and structural characterization of polypeptide antibiotics of the peptaibol class by high-performance liquid chromatography with field desorption and fast atom bombardment mass spectrometry. J Chromatogr.

[CR3] Chutrakul C, Alcocer M, Bailey K, Peberdy JF (2008). The production and characterisation of trichotoxin peptaibols, by Trichoderma asperellum. Chem Biodivers.

[CR4] Daniel JFS, Filho ER (2007). Peptaibols of *Trichoderma.* Review. Nat Prod Rep.

[CR5] Degenkolb T, Dçhren HV, Nielsen NF, Samuels GJ, Brckner H (2008). Recent advances and future prospects in peptaibiotics, hydrophobin, and mycotoxin research, and their importance for chemotaxonomy of *Trichoderma* and *Hypocrea.* Review. Chem Biodivers.

[CR6] Geraldine AM, Lopes FAC, Carvalho DDC, Barbosa ET, Rodrigues AR, Brandão RS, Ulhoa CJ, Lobo-Junior M (2013). Cell wall-degrading enzymes and parasitism of sclerotia are key factors on field biocontrol of white mold by *Trichoderma* spp. Biol Cont.

[CR7] Harman GE (2006). Overview of mechanisms and uses of *Trichoderma* spp. Phytopathol.

[CR8] Harman GE, Howell CR, Viterbo A, Chet I, Lorito M (2004). *Trichoderma* species – opportunistic, avirulent plant symbionts. Microbiol.

[CR9] Jaworski A, Brückner H (1999). Detection of new sequences of peptaibol antibiotics trichotoxins A-40 by on-line liquid chromatography–electrospray ionization mass spectrometry. J Chromatogr A.

[CR10] Lorito M, Farkas V, Rebuffat S, Bodo B, Kubicek CP (1996). Cell wall synthesis is a major target of mycoparasitic antagonism by *Trichoderma harzianum*. J Bacteriol.

[CR11] Mikkola R, Andersson MA, Kredics L, Grigoriev PA, Sundell N, Salkinoja-Salonen MS (2012). 20-Residue and 11-residue peptaibols fromthe fungus *Trichoderma longibrachiatum* are synergistic in forming Na+/K + -permeable channels and adverse action towards mammalian cells. FEBS J.

[CR12] Mootz HD, Schwarzer D, Marahiel MA (2002). Ways of assembling complex natural products on modular nonribosomal peptide synthetases. ChemBioChem.

[CR13] Mukherjee PK, Horwitz BA, Kenerley CM (2012). Secondary metabolism in Trichoderma – a genomic perspective. Microbiol.

[CR14] O’brien J, Wright GD (2011). An ecological perspective of microbial secondary metabolism. Curr Opin Biotechnol.

[CR15] Peltola J, Ritieni A, Mikkola R, Grigoriev PA, Pócsfalvi G, Andersson MA, Salkinoja-Salonen MS (2004). Biological effects of *Trichoderma harzianum* peptaibols on mammalian cells. Appl Environ Microbiol.

[CR16] Rahaman A, Lazaridis T (2014). A thermodynamic approach to alamethicin pore formation. Biochim Biophys Acta.

[CR17] Rebuffat S, Goulard C, Bodo B (1995). Antibiotic peptides from *Trichoderma harzianum:* harzianins HC, proline-rich 14-residue peptaibols. J Chem Soc Perkin Trans.

[CR18] Reino JL, Guerrero RF, Hernández-Galán R, Collado IG (2008). Secondary metabolites from species of the biocontrol agent Trichoderma. Phytochemi Rev.

[CR19] Ren J, Xue C, Tian L, Xu M, Chen J, Deng Z, Proksch P, Lin W (2009). Asperelines A-F, Peptaibols from the Marine-Derived Fungus *Trichoderma asperellum*. J Nat Prod.

[CR20] Ren J, Yang Y, Liu D, Chen W, Proksch P, Shao B, Lin W (2013). Sequential determination of new peptaibols asperelines G-Z12 produced by marine-derived fungus Trichoderma asperellum using ultrahigh pressure liquid chromatography combined with electrospray-ionization tandem mass spectrometry. J Chromatogr A.

[CR21] Schirmböck M, Lorito M, Wang Y, Hayes CK, Arisan-Atac I, Scala F, Harman GE, Kubicek CP (1994). Parallel formation and synergism of hydrolytic enzymes and peptaibol antibiotics, molecular mechanisms involved in the antagonistic action of *Trichoderma harzianum* against phytopathogenic fungi. Appl Environ Microbiol.

[CR22] Schwarzer D, Finking R, Marahiel MA (2003). Nonribosomal peptides: from genes to products. Nat Prod Rep.

[CR23] Shuster A, Schmoll M (2010). Biology and biotechnology of *Trichoderma*. Appl Microbiol Biotechnol.

[CR24] Stoppacher N, Neumann NK, Burgstaller L, Zeilinger S, Degenkolb T, Brückner H, Schuhmacher R (2013). The comprehensive peptaibiotics database. Chem Biodivers.

[CR25] Suwan S, Isobe M, Kanokmedhakul S, Lourit N, Kanokmedhakul K, Soytong K, Koga K (2000). Elucidation of high micro-heterogeneity of an acidic-neutral trichotoxin mixture from Trichoderma harzianum by electrospray ionization quadrupole time-of-flight mass spectrometry. J Mass Spectrom.

[CR26] Vinale F, Sivasithamparam K, Ghisalberti EL, Marra R, Woo SL, Lorito M (2008). *Trichoderma*–plant–pathogen interactions. Soil Biol Biochem.

[CR27] Vinale F, Sivasithamparam K, El G, Ruocco M, Woo S, Lorito M (2012). *Trichoderma* secondary metabolites that affect plant metabolism. Nat Prod Commun.

[CR28] Wang HN, Shi M, Xie ST, Luo Y, Sun CY, Chen XL, Zhang YZ (2010). Antimicrobial peptaibols, novel suppressors of tumor cells, targeted calcium-mediated apoptosis and autophagy in human hepatocellular carcinoma cells. Mol Cancer.

